# Minimally invasive approach for retrorectal tumors above and below S3: a multicentric tertiary center retrospective study (MiaRT study)

**DOI:** 10.1007/s10151-024-02938-y

**Published:** 2024-06-11

**Authors:** T. Bardol, R. Souche, C. Druet, M. M. Bertrand, C. Ferrandis, M. Prudhomme, F. Borie, J.-M. Fabre

**Affiliations:** 1 Digestive and Minimally Invasive Surgery Unit, Montpellier University Hospital, University of Montpellier-Nîmes, 641 Avenue du Doyen Gaston Giraud, 34090 Montpellier, France; 2 Department of Digestive and Oncological Surgery, Nîmes University Hospital, Montpellier-Nîmes University, Montpellier, France

**Keywords:** Retrorectal tumors, Tailgut cyst, Minimally invasive surgery, Laparoscopy: TAMIS

## Abstract

**Background:**

Retrorectal tumors are uncommon lesions developed in the retrorectal space. Data on their minimally invasive resection are scarce and the optimal surgical approach for tumors below S3 remains debated.

**Methods:**

We performed a retrospective review of consecutive patients who underwent minimally invasive resection of retrorectal tumors between 2005 and 2022 at two tertiary university hospital centers, by comparing the results obtained for lesions located above or below S3.

**Results:**

Of over 41 patients identified with retrorectal tumors, surgical approach was minimally invasive for 23 patients, with laparoscopy alone in 19, with transanal excision in 2, and with combined approach in 2. Retrorectal tumor was above S3 in 11 patients (> S3 group) and below S3 in 12 patients (< S3 group). Patient characteristics and median tumor size were not significantly different between the two groups (60 vs 67 mm; *p* = 0.975). Overall median operative time was 131.5 min and conversion rate was 13% without significant difference between the two groups (126 vs 197 min and 18% vs 8%, respectively; *p* > 0.05). Final pathology was tailgut cyst (48%), schwannoma (22%), neural origin tumor (17%), gastrointestinal stromal tumor (4%), and other (19%). The 90-day complication rates were 27% and 58% in the > S3 and < S3 groups, respectively, without severe morbidity or mortality. After a median follow-up of 3.3 years, no recurrence was observed in both groups. Three patients presented chronic pain, three anal dysfunction, and three urinary dysfunction. All were successfully managed without reintervention.

**Conclusions:**

Minimally invasive surgery for retrorectal tumors can be performed safely and effectively with low morbidity and no mortality. Laparoscopic and transanal techniques alone or in combination may be recommended as the treatment of choice of benign retrorectal tumors, even for lesions below S3, in centers experienced with minimally invasive surgery.

**Supplementary Information:**

The online version contains supplementary material available at 10.1007/s10151-024-02938-y.

## Introduction

Retrorectal tumors are a group of heterogeneous and rare lesions. These tumors are in the retrorectal space limited anteriorly by the rectum, posteriorly by the sacrum (Waldeyer fascia), by the peritoneal reflection superiorly, the levator ani and coccygeus muscles inferiorly. The actual incidence has been estimated to be approximately one case per 40,000 admissions. It is most often a benign tumor that affects young women, and the origin is congenital in 60% of cases [1].

Uhlig and Johnson classification is the most used to classify retrorectal tumors [2]. The most common benign lesion is a tailgut cyst which is a hamartoma arising from remnants of the embryological postanal primitive gut [3]. Chordoma is the most common malignant lesion that classically affects middle-aged men [1].

Retrorectal tumors are most often asymptomatic or paucisymptomatic. Consequently, the diagnosis is regularly made accidentally after a morphological examination such as an abdominal ultrasound or a CT scan. MRI is the gold standard morphological exam to determine the structure of the lesion, its origin, its topography, and its extension to adjacent organs [4, 5]. All these parameters are essential to define the type of surgery and its approach.

When a retrorectal tumor is diagnosed, complete surgical excision is the rule especially in case of a malignant lesion [6–8]. A biopsy is not helpful if there is no suspicion of a degenerate lesion or no plan to initiate neoadjuvant therapy. Usually, lesions located below the third sacral vertebra (S3) are approached by dorsal transsacrococcygeal, perineal or combined approach (abdominal and perineal approach) while for those located above S3 the approach is transabdominal (open or minimally invasive) [4]. In most cases these lesions are benign and limiting surgical morbidity is fundamental (especially long-term functional consequences).

On the basis of our experience in minimally invasive surgery and in particular in retrorectal tumors, laparoscopy has become our first-line approach regardless of the location of the lesion compared to S3 [9]. Our hypothesis is that minimally invasive surgery (MIS) is reliable, safe, and allows one to obtain satisfying histopathological results while limiting postoperative morbidity and functional sequelae independently of tumor location.

## Materials and methods

### Study design

We reviewed all patients who underwent surgery for a retrorectal tumor (RRT) at two tertiary university hospital centers between 2005 and 2022. We conducted a retrospective review of prospectively collected medical record data pertaining to patients. This data encompassed individuals who presented with RRTs, characterized as solitary lesions situated in the extraperitoneal retrorectal space.

All cases were discussed in a multidisciplinary team meeting with the presence of, at least, a radiologist, an oncologist, a gastroenterologist, and a digestive surgeon pre- and postoperatively. Surgical approach was defined according to the location of the inferior tumor pole with respect of S3, the tumor size, and morphology. All tumors above S3 were approached laparoscopically. In case of intraoperative difficulties, transanal minimally invasive surgery (TAMIS) was associated to achieve complete resection. Finally, TAMIS approach alone was proposed for small (< 40 mm), unilocular RRT located below S3.

We systematically documented patients’ demographic information, clinical symptoms, and diagnostic methodology. We also collected documented several surgical parameters, including the operative duration, hospital length of stay, and immediate postoperative morbidity classification based on the Clavien–Dindo classification [10]. All participants received annual follow-up assessments, accompanied by either MRI or CT scans. This study was ethically approved by our institutional review board (IRB) and registered on ClinicalTrials.gov (RECHMPL21_0093; NCT04757103).

### Surgical procedures

All patients underwent minimally invasive surgical procedures: laparoscopic, TAMIS, or combined approach. After general anesthesia, patients were placed in the Trendelenburg position with legs spread apart authorizing a transanal approach if necessary. Laparoscopic surgical procedures (Supplementary videos 1 and 2) were performed as previously described [9]. TAMIS procedures were achieved using GelPOINT® Path Transanal Access Platform (Applied Medical, USA) as shown in Supplementary video 3. After a Lone Star® retractor system was introduced into the anal canal, a digital rectal examination allowed the identification of the lesion and the GelPOINT® Platform (Applied Medical, USA) was inserted. Once the tumor was palpated, the posterior rectal wall was opened, reaching the retrorectal infraperitoneal space, and the lesion was therefore identified. Step-by-step dissection was performed using monopolar energy and/or Thunderbeat® (Olympus, USA) device respecting the levator ani muscle and the coccyx. Complete excision of the lesion was achieved and referred for histological examination. Finally, closure of the posterior rectal wall was performed using a resorbable barbed suture V-loc™ (Medtronic, UK) without prophylactic drainage. Bladder catheter was removed the day after the surgical procedure and patients were discharged once the bowel function was obtained.

### Statistical analysis

Numeric variables were expressed as mean (± SD) and discrete outcomes as absolute and relative (%) frequencies. We created two groups according to the location of the inferior tumor pole (below or above S3). Normality and heteroskedasticity of data were assessed with the Shapiro–Wilk test and Levene’s test. Continuous outcomes were compared with unpaired Student *t* test, Welch *t* test, or Mann–Whitney *U* test according to data distribution. Discrete outcomes were compared with chi-squared or Fisher’s exact test accordingly. The alpha risk was set to 5% and two-tailed tests were used. Statistical analysis was performed with EasyMedStat (version 3.30; www.easymedstat.com).

## Results

**Fig. 1:**
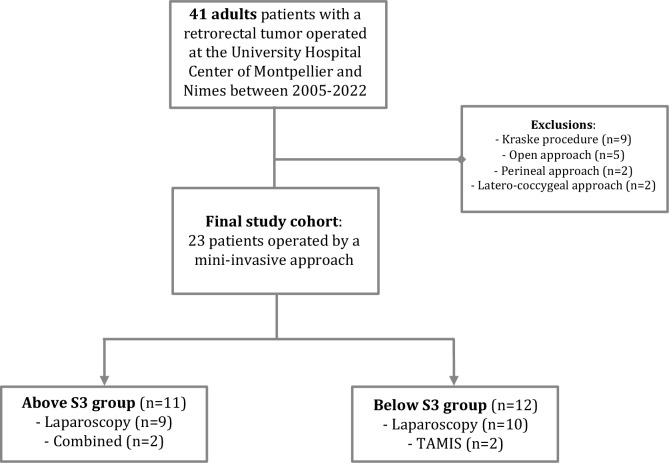
MiaRT study flowchart

Forty-one patients were operated on during the study period. After exclusion criteria were applied, 23 patients who underwent a minimally invasive approach were included and analyzed. These patients were divided into two groups according to the localization of the lower pole of the tumor regarding the third sacral vertebra (Fig. 1). Women represented most of the cohort (*n* = 20, 87%) with a median age of 42 years old (range 21–72). Only one patient was evaluated with an American Society of Anesthesiologists (ASA) score of 3. Ten out of 23 patients had an history of previous abdominal surgery (43%). All demographic data are summarized in Table 1. The two groups were comparable in terms of demographic data and clinical symptoms (all *p* > 0.05).

**Table 1:** Demographic and preoperative data

Variables	Total	Above S3	Below S3	*P*
Patients	23	11	12	
Gender				0.217
Female	20 (87)	11 (100)	9 (75)	
Male	3 (13)	0	3 (25)	
Age (years), median (range)	42 (21–72)	44 (21–72)	40 (19–62)	0.573
BMI (kg/m^2^), median (range)	23.1 (17.2–28.7)	23.2 (18–28)	23.6 (17.2–28.7)	0.785
ASA score				0.667
Grade I	14 (61)	7 (64)	7 (58)	
Grade II	8 (35)	3 (27)	5 (42)	
Grade III	1 (4)	1 (9)	0	
Grade IV	0	0	0	
Comorbidities				
High blood pressure	3 (13)	1 (9)	2 (17)	> 0.999
Cardiovascular disease	1 (4)	1 (9)	0	0.478
Smoker	7 (30)	3 (27)	4 (33)	> 0.999
Respiratory disease	1 (4)	0	1 (8)	> 0.999
Diabetes mellitus	1 (4)	0	1 (8)	> 0.999
Previous abdominal surgery	10 (43)	6 (54)	4 (33)	0.414
Preoperative symptoms^a^				> 0.999
Abdominal pain	10 (43)	5 (45)	5 (42)	
Diarrhea	2 (9)	1 (9)	1 (8)	
Sciatica	2 (9)	1 (7)	1 (8)	
Dysuria	3 (12)	1 (7)	2 (17)	
Asymptomatic	10 (43)	5 (45)	5 (42)	

Values are number (percentage) unless otherwise indicated

*BMI* body mass index, *ASA* American Society of Anesthesiologists

^a^Two patients had several preoperative symptoms

### Preoperative management

Clinical presentation varies from asymptomatic (incidentaloma) in ten cases (10/23, 43%) to abdominal pain (10/23, 43%) or sciatica in two cases (2/23, 9%). Dysuria and diarrhea were the other reported symptoms (Table 1).

A pelvic MRI was systematically performed for all patients (*n* = 23/23, 100%) and more than one of two patients had an abdominopelvic CT scan (13/23, 56%). None of them had a preoperative biopsy (Table 2).

**Table 2:** Retrorectal tumor characteristics

Variables	Total (*n* = 23)
Morphologic features	
Tumor size (mm), median (range)	60 (30–200)
Tumor morphology	
Unilocular	13 (56)
Multilocular	7 (30)
Not reported	3 (13)
Solid component	8 (35)
Lower tumor pole localization	
Above S3	11 (48)
Below S3	12 (52)
Preoperative workup	
US	5 (22)
CT scan	13 (56)
MRI	23 (100)
EUS	1 (4)
PET scan	1 (4)
Preoperative biopsy	0

Values are number (percentage) unless otherwise indicated

*S3* indicates third sacral vertebra, *SU* ultrasound, *CT* computed tomography, *MRI* magnetic resonance imaging, *EUS* endoscopic ultrasound

### Surgical and histological data

Among 23 patients who underwent minimally invasive surgical procedures, 19 had a pure laparoscopic approach, 2 had TAMIS, and 2 had a combined approach. Amongst them 12 patients had an inferior tumor pole located below the third sacral vertebra (S3) (12/23, 52%). The median tumor size was not significantly different between the two groups (60 vs 67 mm; *p* > 0.999). Thirteen were unilocular tumors (13/23, 56%) and 35% of the retrorectal tumors (8/23) had a solid component on the preoperative MRI. There was no significant difference in terms of tumor characterization (morphology and presence of solid component) between the two groups (all *p* > 0.05).

All lesions located above S3 were removed by laparoscopic approach alone in nine patients (9/11, 82%) and combined with a TAMIS procedure in the other two cases (2/11, 18%). In the “below S3” group, 83% of excision were achieved by laparoscopic approach (10/12). There was no significant difference in terms of conversion to open surgery between the two groups (*p* = 0.322).

Forty-eight percent of the resected RRT were tailgut cysts (11/23). The other final pathology was schwannoma (5, 22%), neurinoma (2, 9%), GIST (1, 1%), myelolipoma (1, 4%), ganglioneuroma (1, 4%), anterior meningocele (1, 4%), paraganglioma (1, 4%), and dermoid cyst (1, 4%). Final histology of resected RRT was significantly different between the two groups with a predominance of tailgut cysts in the below S3 group compared to the above S3 group (*p* = 0.002). Surgical and pathological characteristics are summarized in Table 3.

**Table 3:** Surgical and histological features

Variables	Above S3	Below S3	*P*
	(*n* = 11)	(*n* = 12)	
Surgical approach			0.23
Laparoscopic	9 (82)	10 (83)	
Transanal (TAMIS)	0	2 (17)	
Combined	2 (18)	0	
Intraoperative features			
Duration of surgery (min), median (range)	126 (29–292)	197 (80–298)	0.271
Intraoperative blood loss (ml), median (range)	25 (0–100)	50 (0–500)	0.859
Drainage	4 (36)	3 (25)	0.667
Conversion to open approach	2 (17)	1 (8)	0.322
Final histology			**0.002**
Tailgut cyst	2 (18)	9 (76)	
Dermoid cyst	0	1 (8)	
Schwannoma	5 (45)	0	
Neurinoma	2 (18)	0	
Ganglioneuroma	0	1 (8)	
Meningocele	0	1 (8)	
Myelolipoma	1 (9)	0	
GIST	1 (9)	0	
Resection margin			> 0.999
R0	10 (91)	11 (92)	
R1	1 (9)	1 (8)	

Values are number (percentage) unless otherwise indicated

*TAMIS* transanal minimally invasive surgery

### Outcome

No major postoperative complication (Clavien–Dindo ≥ 3) or death was reported in either group (Table 4). The median hospital stay was 4 days (range 2–9). Most of the reported postoperative symptoms were sciatica (4/23, 17%), urinary retention (3/23, 13%), and locoregional dysesthesia (3/23, 13%). The location of the tumor was not associated with the occurrence of such complications. Chronic pain was reported in four cases of the entire cohort whereas urinary or fecal dysfunction were reported in patients with retrorectal tumors a below S3. During the follow-up (median 3.3 years), no recurrence was diagnosed in either group.

**Table 4:** Postoperative outcomes after minimally invasive excision of retrorectal tumors

Variables	Above S3	Below S3	*P*
	(*n* = 11)	(*n* = 12)	
30-day postoperative morbidity			
Severe complications (Clavien–Dindo ≥ 3)	0	0	1
Sepsis	0	0	1
Reoperation	0	0	1
Urinary symptoms	0	3 (25)	0.217
Sciatica	3 (27)	1 (8)	0.316
Locoregional hypoesthesia	2 (18)	1 (8)	0.590
LOS (days), median (range)	4 (2–5)	4.7 (3–9)	0.462
90-day postoperative morbidity			
Fecal dysfunction	0	3 (25)	0.217
Urinary dysfunction	0	3 (25)	0.217
Sexual dysfunction	0	0	1
Chronic pain	3 (27)	1 (8)	0.316
90-day postoperative mortality	0	0	1

Values are number (percentage) unless otherwise indicated

*LOS* length of stay

## Discussion

This study aimed to assess the safety and feasibility of a MIS approach for retrorectal tumors regardless of tumor location. Here we report a cohort of 23 consecutive patients operated on with a minimally invasive approach regardless of the location of the inferior tumor pole in two tertiary centers with an expertise in minimally invasive procedures and especially excision of retrorectal tumors [9, 11–13]. In the present cohort, the success rate of transabdominal laparoscopic approach is 82.6%. This systematic MIS strategy was associated with encouraging results for tumors regardless of the location of the inferior tumor pole.

No serious complications occurred during surgery and severe postoperative morbidity was nil in the two groups. Also, median operative time was not significantly different between the two groups. Conversely, patients were discharged on postoperative day 4. Those results are concordant with recently published series of resected RRT [14, 15]. It is noteworthy, to observe that there were three cases of conversion to open surgery without significant difference between the > S3 and < S3 groups. This 13% conversion rate is higher than the rate reported by Galán et al. but similar to the results published by Aubert et al. [14, 15]. However, Aubert et al. reported a conversion rate of 19% for the laparoscopic approach [14]. This relative high conversion rate could be explained by a large median tumor size (60 mm). The median tumor size was even larger (75 mm) in patients who needed a conversion to open surgery. Secondly, our systematic MIS approach could also explain this conversion rate regardless of tumor size and history of previous abdominal surgery (43% rate in our cohort). While this approach may be considered a limitation in this case, our results show that lesions below S3 can also be successfully resected through MIS. Indeed, only one conversion was reported in the below S3 group.

In our study, we report four cases of chronic pain (17%) and three cases of urinary and fecal dysfunctions at 90 days postoperatively. Those results are in line with previous publications [1]. For example, a comprehensive literature review by Baek et al. found that the rate of postoperative complications in patients who underwent surgery for retrorectal tumors was 13%. The authors reported a 23% rate of neurogenic bladder and 18% of neurologic complications including pain [1]. Aubert et al. also reported a 17% rate of chronic pain after RRT excision without significant difference according to the surgical approach (anterior or posterior) [14]. Our results suggest that MIS is safe for the surgical management of RRT even below S3.

Long-term surveillance of RRTs is mandatory to monitor potential recurrence. Typically, local recurrence rates are lower for benign tumors (1–2%) than for malignant tumors (30–50%) [16]. In our study, no recurrence was observed after a median follow-up of more than 3 years mainly because final pathology exams did not reveal any malignant lesion.

The retrospective design of the study is the first limitation with possible selection and confusion biases. Indeed, the absence of malignant lesion after pathology exam in our cohort reflects the expertise of our radiological department and our intention to develop a personalized surgical approach. However, this limitation mitigates the results of our study and prevents us from recommending MIS approach for suspected or confirmed malignant RRT. Also, our study is constrained by the limited number of cases, owing to the rarity of RRTs. To ascertain the long-term effectiveness of the laparoscopic approach to RRT, future investigations employing a multicenter prospective design may offer valuable insights.

If the laparoscopic approach is now considered as the standard of care in rectal cancer surgery, this is only the case for RRT located above S3 [14, 17]. Nonetheless, the progress in MIS techniques has enabled the refinement of surgical strategies for very low rectal cancer lesions. Mullaney et al. reviewed all the RRT excised by a MIS approach and reported only nine patients who received a robotic-assisted surgery. Median tumor size range from 5 to 10 cm and eight patients were operated on for a benign lesion. However, localization regarding the S3 was not reported [18]. Hence, the use of a robotic-assisted approach seems feasible, safe, and associated with minimal postoperative complications and a short hospital stay.

**Fig. 2:**
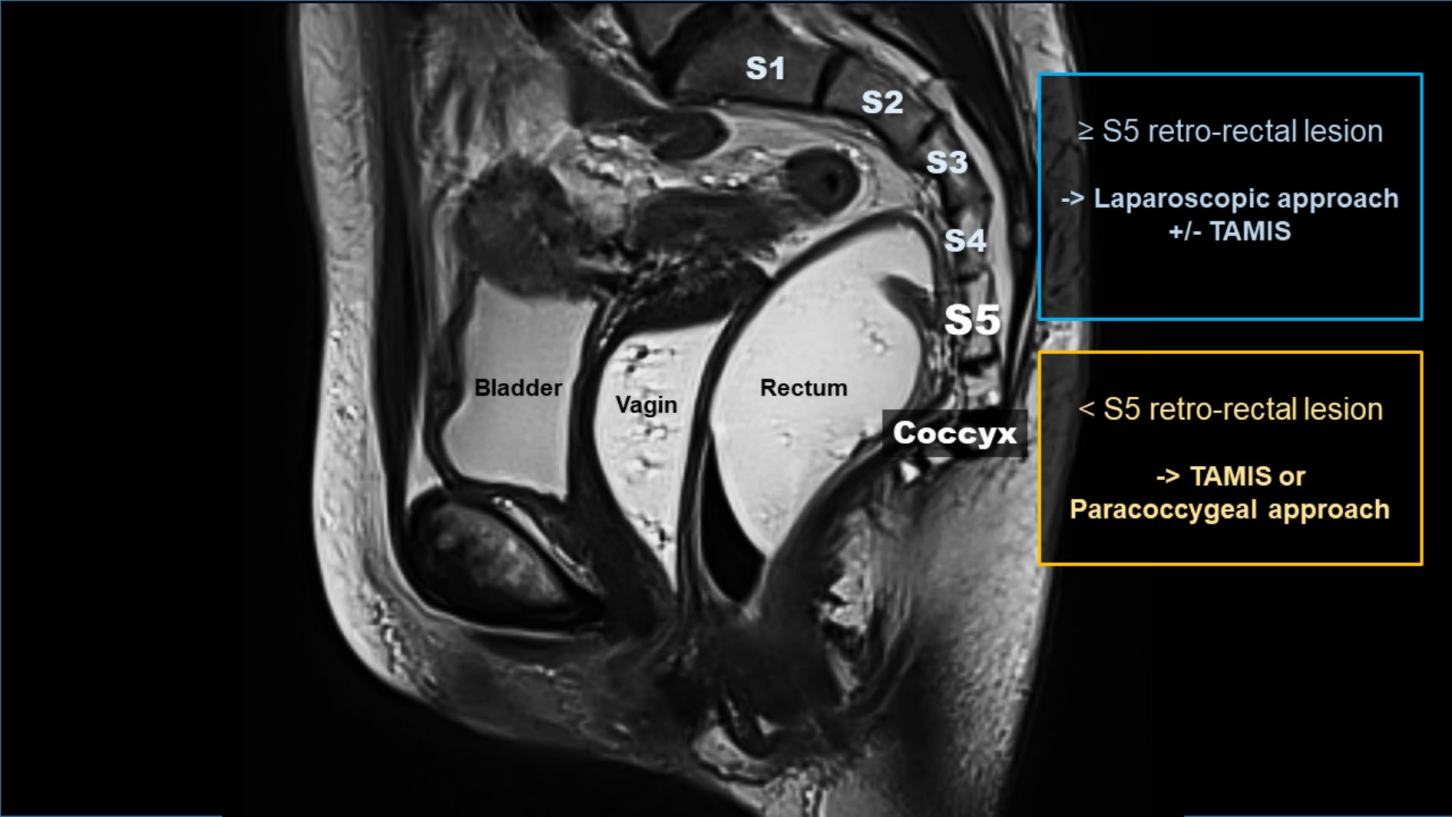
Tailored surgical practice algorithm for retrorectal tumors

In our clinical practice, we considered a laparoscopic transabdominal approach for all RRT above S3 as recommended by Woodfield et al. [4]. TAMIS approach alone or a paracoccygeal approach (modified Kraske procedure) was reserved for smaller lesions and those closer to the pelvic floor. Drawing from the findings of our research, we propose a paradigm shift by using S5 as a new divisor. Moreover, we think that the ongoing spread of robotic-assisted procedures will favor this paradigm shift (Fig. 2).

On the basis of our study results, we cannot recommend a systematic MIS approach in cases where malignancy is suspected, especially when neighboring structures are compromised. It remains crucial to personalize the surgical strategy according to the patient and the tumor configuration with the main goals to prevent tumor wall rupture and obtain complete tumor resection.

## Conclusion

Minimally invasive surgery for retrorectal tumors can be performed safely and effectively with minimal morbidity, zero mortality, and no recurrence. Transabdominal laparoscopy and TAMIS techniques, either individually or in combination, may be recommended for managing benign retrorectal tumors, including those situated above S5, especially when administered by experienced minimally invasive surgery centers.

## Supplementary Information

Below is the link to the electronic supplementary material.

Supplementary file1 (MOV 435300 KB)

Supplementary file2 (MOV 211772 KB)

Supplementary file3 (MP4 396272 KB)

## Data Availability

Data will be made available on request.
